# Atypical antipsychotic therapy in Parkinson's disease psychosis: A retrospective study

**DOI:** 10.1002/brb3.639

**Published:** 2017-04-14

**Authors:** Mei Yuan, Laura Sperry, Norika Malhado‐Chang, Alexandra Duffy, Vicki Wheelock, Sarah Farias, Kevin O'Connor, John Olichney, Kiarash Shahlaie, Lin Zhang

**Affiliations:** ^1^ Department of NeurologyThe Second Affiliated HospitalUniversity Of South ChinaHengyangHunanChina; ^2^ Department of NeurologyUC Davis Medical CenterSacramentoCAUSA; ^3^ Center for Neuroscience and Department of Neurobiology, Physiology and BehaviorUC Davis Medical CenterUC DavisSacramentoCAUSA; ^4^ Department of Neurologic SurgeryUC Davis Medical CenterSacramentoCAUSA

**Keywords:** adverse drug reactions, antipsychotics, Parkinson's disease, Parkinson's disease psychosis

## Abstract

**Objective:**

Parkinson's disease psychosis (PDP) is a frequent complication of idiopathic Parkinson's disease (iPD) with significant impact on quality of life and association with poorer outcomes. Atypical antipsychotic drugs (APDs) are often used for the treatment of PDP; however, their use is often complicated by adverse drug reactions (ADRs). In this study, we present patients with PDP who were treated with the most commonly used atypical antipsychotic agents and review their respective ADRs.

**Methods:**

A retrospective study was carried out to include a total of 45 patients with iPD who visited a movement disorders clinic between 2006 and 2015. All PDP patients treated with atypical APDs were included in the analysis for their specific ADRs.

**Results:**

Forty‐five iPD patients (mean age of onset: 62.67 ± 9.86 years) were included, of those 10 patients had psychosis (mean age of onset: 76.80 ± 4.61 years). Of the 45 patients, 22.2% were found to have psychotic symptoms, of whom 70% had hallucinations, 20% had delusions, and 10% illusions. Seventy percent of psychotic symptoms occurred after ten or more years from diagnosis of iPD. PDP patients were treated with quetiapine, olanzapine, and risperidone separately or in combination, all of which were found to have certain ADRs.

**Limitations:**

This study was limited by its retrospective study design and small sample size and with likely selection bias.

**Conclusions:**

The prevalence of PDP is relatively high in older patients with iPD. The uses of the currently available atypical APDs in this patient population are often complicated by ADRs. The selective 5‐HT_2A_ inverse agonist, pimavanserin, could be a better alternative in the treatment of PDP.

## 1 Introduction

Parkinson's disease (PD) is a slowly progressive neurodegenerative disease that affects 7–10 million people worldwide and is more common in older people. The number of PD patients is expected to increase in parallel to the rising age of our population. PD is characterized by a progressive loss of motor function and a wide spectrum of nonmotor symptoms (NMSs). NMSs in PD have been systematically described and include a high prevalence of neuropsychiatric symptoms, such as psychosis, depression, cognitive impairment, and sleep disturbances (Munhoz, Moro, Silveira‐Moriyama, & Teive, 2015). Psychosis in PD, also known as Parkinson's disease psychosis (PDP), can affect more than 50% of people with PD and result in a significant impact on patients’ quality of life (Starkstein, Brockman, & Hayhow, 2012). In addition, symptoms of PDP are associated with increased caregiver burden, greater likelihood of placement in nursing homes and increased mortality.

PDP is characterized by hallucinations (particularly visual), delusions, illusions and false sense of presence. The etiology of PDP is thought to be multifactorial. Some studies show that the development of PDP is associated with the use of dopaminergic medications (Bizzarri et al., 2015). However, other studies suggest medications alone cannot explain emergence of psychosis (including no dose relationship and present in newly diagnosed patients (Pagonabarraga et al., 2016). There are also some studies suggest that increased age, male gender, lower levels of education, older age at onset, longer disease duration and serious medical conditions also contribute to the development of PDP (Gama et al., 2015; Zhu, van Hilten, Putter, & Marinus, 2013).

What is also noteworthy is that the pathophysiology of PDP is most likely attributed to disorders of the cholinergic and serotonergic systems (Mocci et al., 2014; Rolland et al., 2014) as well as to changes in brain structure (Kiferle et al., 2014). With regard to therapeutics, PDP can be difficult to treat due to the lack of safe, effective pharmacological treatments. The first‐line strategy in the treatment of persistent and problematic PDP is represented by reduction in anti‐PD medications. However due to different and multiple factors and unclear pathophysiology, it does not always work well. Besides, reducing anti‐PD medications may aggravate PD symptoms. Antipsychotics are usually prescribed off‐label (Goldman & Holden, 2014; Seppi et al., 2011). Compared with first‐generation antipsychotics or typical antipsychotics, second‐generation antipsychotics (SGAs) produce fewer adverse effects on motor function and serum prolactin elevation, presumably due to dual serotonin‐dopamine antagonism (Noel, 2007). As a result, SGAs, also known as atypical antipsychotic drugs (APDs) are frequently prescribed for patients with PDP.

The search for effective strategies for the treatment of PDP has been an area of much interest. The only drug with confirmed benefit without worsening Parkinsonism has been clozapine (Seppi et al., 2011). However the avoidance of clozapine in large part due to the inconvenience of frequent blood testing (Hack et al., 2014). Additionally, patients and providers must both be registered with the FDA for clozapine to be prescribed. For these reasons, APDs such as quetiapine, risperidone, and olanzapine are more commonly used. In this article, we review patients with PDP who were treated with these three atypical antipsychotic drugs separately or in combination over the past 10 years in an outpatient specialty clinic and their adverse drug reactions (ADRs).

## 2 Material and Methods

A retrospective chart review of electronic medical records (EMRs) was performed on 445 patients who visited our movement disorders clinic at UC Davis Medical Center between January 2006 and December 2015. Because data were collected retrospectively from records and the identity of patients was not obtained, informed consent was not required. After reviewing each patient's EMR, all patients were screened for a diagnosis of iPD with supportive features on a complete neurological examination including muscle tone, posture, gait and involuntary movement. Besides, iPD patients with incomplete records and/ or with psychotic symptoms attributable to other conditions were excluded. Demographic variables collected included sex, age range, and age at onset of PD and age at onset of PDP. Clinical variables include antiparkinsonian treatment, APD use and ADRs, and symptoms consistent with psychosis. On the basis of our systemic chart review, we were able to confirm the diagnosis of iPD in 45 of 445 patients, using the United Kingdom Parkinson's Disease Society Brain Bank (UKPDSBB) diagnostic criteria. Ten of 45 patients with iPD were included in this report that met the diagnostic criteria for psychosis in Parkinson's disease published in 2007 and updated in 2013.

Data were reported as simple statistics, including number, percentage, mean, and standard deviation. We used constituent ratios (CR) to evaluate different psychotic symptoms in patients with PDP. SPSS 13.0 was used to compute the mean and standard deviation.

## 3 Results

Demographic and clinical characteristics of iPD patients are shown in Table 1. Forty‐five iPD patients (mean age of onset: 62.67 ± 9.86 years) were included; of those, 10 patients (22.2%) had symptoms of psychosis (mean age of onset: 76.80 ± 4.61 years). The psychotic symptoms included hallucinations (70%), delusions (20%), and illusions (10%). All hallucination reported among our cohort were visual hallucinations. Descriptions of visual hallucinations included seeing people and fire or dots of light in the peripheral fields. Additionally, two patients reported paranoid delusions. One of them thought that people were planning on harming him while the other patient thought that her husband was having an extramarital affair. The patient who experienced an illusion noted that the table appeared to move like a dog. Psychotic symptoms occurred up to several times per day and mostly while alone, or in a quiet environment.

**Table 1:** Demographic and clinical characteristics of iPD patients

Measurement	Mean ± SD or %
Age (years)	75.87 ± 10.01
Males (%)	28/45 (62.2)
Age at onset of PD (years)	62.67 ± 9.86
Age at onset of PDP (years)	76.80 ± 4.61
L‐dopa monotherapy, *n* (%)	21 (46.7)
L‐dopa + agonist, *n* (%)	14 (31.1)
Agonist monotherapy, *n* (%)	4 (8.9)
Other type of antiparikinson drugs	6 (15.3%)
PDP, *n* (%)	10/45 (22.2)
Hallucinations, *n* (%)	7/10 (70)
Delusions, *n* (%)	2/10 (20)
Illusion, *n* (%)	1/10 (10)

iPD, idopathic Parkinson's disease; PDP, Parkinson's disease psychosis; SD, standard deviation.

In our report, there were 46.7% iPD patients exposed to L‐dopa monotherapy and 23.8% of these patients were reported to develop PDP. Of those taking L‐dopa and dopamine agonist therapy together, 28.6% developed psychosis. Twenty‐five percent reported onset of psychosis during dopamine agonist therapy alone (Table 2). None of the six patients on nondopaminergic drugs for PD developed psychosis.

**Table 2:** The percentage of iPD patients developed PDP after taking antiparkinsonian drugs

Antiparkinsonian drugs	Number of iPD	Number of PDP	Percentage (%)
L‐dopa monotherapy	21	5	23.8
L‐dopa + Dopamine agonist	14	4	28.5
Dopamine agonist monotherapy	4	1	25
Other type of antiparikinson drugs	6	0	0

iPD, idopathic Parkinson's disease; PDP, Parkinson’ s disease psychosis.

Psychotic symptoms mainly occurred ten or more years after the diagnosis of iPD. Psychotic symptoms were observed in 10% of patients within 5 years of diagnosis and in 80% of patients within 15 years of diagnosis. One‐half occurred in the interval of 10 to 15 years after the diagnosis of iPD (Table 3). We also found that the male patients with iPD constituted 60% of incident psychosis cases, with a male to female ratio of 3:2 (Table 3).

**Table 3:** Onset of PDP since diagnosis of iPD (in years)

Sex	Years				Total, *n* (%)
	<5	[5–10]	[10–15]	>15	
Male (*n*)	1	2	3	0	6 (60)
Female (*n*)	0	0	2	2	4 (40)
Total, *n* (%)	1 (10)	2 (20)	5 (50)	2 (20)	10 (100)

iPD, idopathic Parkinson's disease; PDP, Parkinson's disease psychosis.

The ten PDP patients we reviewed were treated with quetiapine, olanzapine and/or risperidone separately or in combination. Six patients reported mild improvement of psychosis when treated with quetiapine, whereas one patient had no benefit from it. Two patients received olanzapine monotherapy and both had improvement in psychotic symptoms. Two patients were treated with risperidone: one received risperidone monotherapy, whereas the other patient was treated with quetiapine initially, and then switched to risperidone. Both experienced alleviation of psychotic symptoms. However, all atypical APDs used were associated with ADRs (Table 4). Both quetiapine and olanzapine could cause sedation and sialorrhea; both olanzapine and risperidone resulted in worsening of motor symptoms. The daily dose range of quetiapine, olanzapine, and risperidone was 25 mg/day–150 mg/day, 2.5 mg/day–5 mg/day, and 1 mg/day, respectively (Table 4).

**Table 4:** Adverse drug reactions of different antipsychotics

Drug	Number of patients taking atypical APDs	Dosage (mg/day)	ADRs
Quetiapine	7	25–150	Sedation, sialorrhea
Olanzapine	2	2.5–5	Motor worsening, sedation, sialorrhea
Risperidone	2	1	Motor worsening

iPD, idopathic Parkinson's disease; PDP, Parkinson's disease psychosis; SD, standard deviation; ADRs, adverse drug reactions; APDs, Antipsychotic drugs.

## 4 Discussion

With a shift in our understanding of Parkinson's disease over the past two decades, we have developed a greater appreciation for the NMS associated with iPD including significant psychotic symptoms such as hallucinations. Various clinical features of hallucinations have been described with a prevalence that varies widely from 17 to 72% amongst PDP patients (Meral et al., 2007). Consistent with previous studies, our current report also found that visual hallucinations are the most commonly reported psychotic symptom among PDP patients with a prevalence of 70% among our cohort.

In our study, these psychotic symptoms were observed in 70% of patients between 5 to 15 years after receiving a clinical diagnosis of PD, with one‐half occurring 10 to 15 years post diagnosis. We also found that 60% of PDP patients were males, but with a similar incidence of psychotic symptoms to females, considering that 62.2% of our PD patients are male. While a recent study has shown that older age at onset and female sex were associated with an increased risk of hallucinations through cross‐sectional analyses of baseline data and longitudinal analyses of follow‐up data (Fernandez, Lapane, Ott, & Friedman, 2000), there is no consensus whether men or women are more likely to suffer from PDP. After all, our samples are small.

Important risk factors associated with the development of psychotic symptoms in iPD are older age, sex, longer disease duration, and severity. Additionally, previous studies have identified that dopaminergic medication treatment is associated with increased risk of developing hallucinations (Fénelon & Alves, 2010; Reichmann, 2016), whereas some earlier studies have questioned the causative relationship of dopaminergic medications with PDP. Through our small sample study, we observed that the combination of L‐dopa with dopamine agonists may contribute to the development of PDP most strongly followed by L‐dopa therapy alone. This supports a possible causal relationship between dopaminergic replacement therapy and the development of hallucinations.

Given the high prevalence of PDP and its impact on quality of life, there has been significant interest in optimizing symptom control through the use of available atypical APDs despite an unclear pathophysiology underlying PDP. Approved in 1985, clozapine was the first atypical antipsychotic for PDP, with findings to support therapeutic benefit without worsening of motor function. Despite its demonstrated efficacy, subsequent studies have shown that clozapine has potentially fatal agranulocytosis (requiring frequent blood testing) and myocarditis, in addition to relatively milder side effects including sedation, seizures, sialorrhea, weight gain, and metabolic disturbances (Hack et al., 2014; Thomas & Friedman, 2010). In addition, one recent case report in an elderly woman with PDP showed clozapine may induce fatal neuroleptic malignant syndrome (NMS) (Mesquita & Siva, 2014). Moreover, frequent pharmacy visits is another limit factor. Because of these substantial side effects with clozapine therapy, it is often avoided by clinicians. By comparison, quetiapine, olanzapine, and risperidone are proportionally more commonly used.

Quetiapine, another first‐line treatment for PDP, is similar to clozapine in chemical structure. Quetiapine does not require monitoring for blood dyscrasias and has less impact on motor symptoms. Because of no aggravation of motor symptoms and relatively easy to use, it is currently considered the most frequently used antipsychotic drug in PD patients. Nevertheless, patients with PDP who are exposed to quetiapine may develop certain adverse reactions, such as sedation, orthostatic hypotension, dizziness, headache, tachycardia, hypersexuality, constipation, rash, and even neuroleptic malignant syndrome. Besides, there is insufficient evidence for the efficacy of quetiapine. Several randomized clinical trials demonstrated no change in psychotic outcomes with quetiapine (Lertxundi et al., 2015; Prohorov, Klein, Miniovitz, Dobronevsky, & Rabey, 2006). In our current report, there were seven PDP patients treated with quetiapine, six of whom had mild improvement of their psychosis, and one had no benefit. This suggests that, to some extent quetiapine improves the psychotic symptoms associated with iPD (Rabey, Prokhorov, Miniovitz, Dobronevsky, & Klein, 2007). Except for sedation and sialorrhea, there were no other adverse drug reactions reported with quetiapine in our cohort.

Two additional atypical APDs used in PDP are olanzapine and risperidone. Most studies reported olanzapine can lead to intolerable motor deterioration even at low doses (Fernandez, Trieschmann, & Friedman, 2003). Our findings are consistent with these findings from previous studies. Moreover, it is remarkable that an analysis of the Spanish Pharmacovigilance Database indicated olanzapine also could produce NMS in PDP patients; likewise, risperidone can dramatically aggravate motor function in some PD patients (Lertxundi et al., 2015). Consequently, the American Academy of Neurology (AAN) does not recommend these two drugs for the treatment of PDP. In our study, four in ten patients who received olanzapine and risperidone had improvement in psychotic symptoms, but both drugs resulted in worsening of motor symptoms. Therefore olanzapine and risperidone should be generally avoided by movement disorders clinicians.

Nonetheless, our findings must be interpreted in light of the inherent limitations of this study.

The study was retrospective with a small sample size and with likely selection bias. Because of this limitation, it is hard to draw firm conclusions with regard to causation/risk factors. A larger sample size would be needed to provide more robust conclusions.

### 4.1 New treatment options

PDP remains a significant therapeutic challenge. The therapeutic options currently in use, as described above, act as antagonists on mesolimbic dopamine receptors, but also have secondary pharmacological effects of blocking dopamine (DA) D2 receptors, and thereby interfere with the effect of dopamine replacement therapy on motor function. In addition, all the medications mentioned in our manuscript for treatment of PDP symptoms have a boxed warning about increased risk of death in elderly people with dementia. Those pose significant clinical challenges. A post hoc analysis from a multicenter, open‐label extension study showed that current antipsychotic medications significantly increased the mortality rate and adverse events in people with PDP (Weintraub et al., 2016). To date, there has not been an agent that can reliably improve the psychotic symptoms without having adverse side effects such as worsening the motor symptoms of PD, or sedation. In light of this dilemma, researchers have looked to a novel selective 5‐HT_2A _and 5‐HT_2c_ with 40 fold less affinity receptor inverse agonist agent, ACP‐103, without dopaminergic, adrenergic, histaminergic, or muscarinic affinity. This agent has been confirmed as a potent, efficacious, orally active 5‐HT_2A_ receptor inverse agonist with a behavioral and pharmacological profile consistent with utility as an antipsychotic agent (Vanover et al., 2006).

The behavioral effects of pimavanserin (formerly ACP‐103) are similar to those of atypical APDs like quetiapine, olanzapine and risperidone, which have appreciable antagonist activities at 5‐HT2A receptors. However, unlike the atypical APDs, pimavanserin lacks DA D2 antagonist activity and thus does not have the same deleterious effect on dopamine replacement therapy and does not worsen motor symptoms (Shotbolt et al., 2009). Moreover, it is particularly interesting to note that pimavanserin has some unique pharmacological properties compared with the first‐ and second‐generation antipsychotics. Pimavanserin not only reduces 5HT2 receptor activity even below baseline levels, without acting on other receptors, but can also induce a pharmacological response opposite to that of an agonist, thus named as a “selective inverse agonist”.

An animal model of PD showed that pimavanserin not only reversed the psychotic behaviors, but did so without worsening motor function or blocking the ability of L‐Dopa to improve motor behavior (McFarland, Price, & Bonhaus, 2011). They also found that pimavanserin had a higher therapeutic ratio than quetiapine or clozapine (Hubbard, Hacksell, & McFarland, 2013). Several studies have demonstrated the clinical benefit of pimavanserin in PDP. A double blind randomized multi‐center dose‐escalation phase 2 study in PDP patients indicated better safety and tolerability of pimavanserin. It also demonstrated the ability to improve PDP without worsening Parkinsonism, causing sedation, agranulocytosis or NMS (Meltzer et al., 2010). Subsequently, a randomized, placebo‐controlled phase 3 trial further supported that pimavanserin benefit patients with PDP for whom few other treatment options exist (Cummings et al., 2014), which was pivotal study that led to its FDA approval. These studies reported ADRs of nausea, vomiting and a small QT interval increase without associated adverse cardiac events. These ADRs were considered to be dose‐limiting and less serious.

Several questions regarding the treatment of psychotic symptoms in iPD remain. As a novel selective 5‐HT_2A_ inverse agonist, pimavanserin may complement or even replace current available treatment. NUPLAZID^™^ (pimavanserin) has been approved by the U.S. Food and Drug Administration on April 29, 2016. However, it will still require considerable postmarketing research and investigation to ensure the sustained safety and efficacy of pimavanserin.

## Conflicts of Interest

The authors declare that they have no conflict of interest.
